# Effect of Monthly Vitamin D Supplementation on Preventing Exacerbations of Asthma or Chronic Obstructive Pulmonary Disease in Older Adults: Post Hoc Analysis of a Randomized Controlled Trial

**DOI:** 10.3390/nu13020521

**Published:** 2021-02-06

**Authors:** Carlos A. Camargo, Les Toop, John Sluyter, Carlene M. M. Lawes, Debbie Waayer, Kay-Tee Khaw, Adrian R. Martineau, Robert Scragg

**Affiliations:** 1 Department of Emergency Medicine, Massachusetts General Hospital, Harvard Medical School, Boston, MA 02114, USA; 2 Department of General Practice, University of Otago, Christchurch 8140, New Zealand; les.toop@otago.ac.nz; 3 School of Population Health, University of Auckland, Auckland 1142, New Zealand; j.sluyter@auckland.ac.nz (J.S.); Carlene.Lawes@waitematadhb.govt.nz (C.M.M.L.); d.waayer@auckland.ac.nz (D.W.); r.scragg@auckland.ac.nz (R.S.); 4 Department of Public Health and Primary Care, University of Cambridge, Cambridge CB2 2QQ, UK; kk101@cam.ac.uk; 5 Institute for Population Health Sciences, Barts and The London School of Medicine and Dentistry, Queen Mary University of London, London E1 2AB, UK; a.martineau@qmul.ac.uk

**Keywords:** asthma, chronic obstructive pulmonary disease, exacerbations, oral corticosteroids, randomized controlled trial, supplement, vitamin D

## Abstract

Randomized controlled trials have suggested that vitamin D supplementation can prevent asthma and chronic obstructive pulmonary disease (COPD) exacerbations. For COPD, the benefit appears to be limited to individuals with baseline 25-hydroxyvitamin D (25OHD) levels <25 nmol/L. We performed a post hoc analysis of data from a randomized, double-blinded, placebo-controlled trial to investigate the effect that monthly, high-dose vitamin D supplementation (versus placebo) had on older adults with asthma and/or COPD. Specifically, we investigated whether vitamin D supplementation prevented exacerbations of these conditions. Participants were randomly assigned either to an initial oral dose of 200,000 IU vitamin D3 followed by 100,000 IU monthly or to placebo, with an average follow-up period of 3.3 years. Among the 5110 participants, 775 had asthma or COPD at the beginning of the study, and were eligible for inclusion in this analysis. Exacerbations were defined by the prescription of a short-burst of oral corticosteroids. The mean age of the participants was 67 years old, and 56% were male. The mean baseline blood 25OHD level was 63 nmol/L; 2.3% were <25 nmol/L. Overall, we found that vitamin D supplementation did not affect the exacerbation risk (hazard ratio 1.08; 95%CI 0.84–1.39). Among those with baseline 25OHD <25 nmol/L, however, the hazard ratio was 0.11 (95%CI 0.02–0.51); *p* for interaction = 0.001. Although monthly vitamin D supplementation had no overall impact on risk of exacerbations of asthma or COPD, we found evidence of a probable benefit among those with severe vitamin D deficiency.

## 1. Introduction

Among older adults, asthma and chronic obstructive pulmonary disease (COPD) are two major obstructive airway diseases that can be difficult to distinguish, and that may coexist as asthma-COPD overlap syndrome [1]. Both are chronic diseases characterized by periodic exacerbations, which are usually triggered by respiratory virus infections [2]. The treatment of asthma and COPD exacerbations is addressed in international management guidelines [3,4]. The guidelines also address interventions (such as inhaled corticosteroids) that aim to prevent exacerbations, which remain a major cause of morbidity, mortality, and increased healthcare costs.

Given the beneficial effect that vitamin D supplementation has on acute respiratory infections (ARI) [5], a growing number of researchers have investigated whether vitamin D supplements could potentially prevent exacerbations of asthma or COPD [6]. In a 2017 individual participant data (IPD) meta-analysis of all known randomized controlled trials (RCTs) worldwide, Jolliffe and colleagues demonstrated that vitamin D supplements can reduce the risk of asthma exacerbations requiring systemic corticosteroids, especially among those with baseline serum hydroxyvitamin D (25OHD) levels <25 nmol/L [7]. In contrast, vitamin D supplementation had no overall effect on the risk of COPD exacerbations among older individuals [8]. However, a prespecified subgroup analysis did show benefits from vitamin D supplements among COPD patients with baseline 25OHD levels of <25 nmol/L. Indeed, all three trials in this IPD meta-analysis reported the same subgroup finding [9,10,11]. A fourth trial with only 88 participants reported an overall protective effect of vitamin D supplementation [12].

Nevertheless, questions remain. We performed a large RCT of monthly high-dose vitamin D supplements (versus placebo) in >5000 older adults in New Zealand. In the current, prespecified analysis, we investigated the effect of vitamin D supplementation on the subgroup of participants with asthma or COPD. Specifically, we investigated whether taking the monthly vitamin D supplement reduced exacerbations overall, or at least in the subgroup with baseline 25OHD <25 nmol/L.

## 2. Materials and Methods

Older adults with asthma or COPD came from a large, population-based RCT of vitamin D supplementation called the Vitamin D Assessment (ViDA) study; this trial was carried out in Auckland, New Zealand. Full details of the study methods [13], and the findings on the main outcomes of cardiovascular disease [14], falls/fractures [15], and ARI [16] have been published. The New Zealand Multi-Region Ethics Committee, Wellington, approved the trial in October 2010 (MEC/09/08/082), and the main outcomes were registered with the Australian New Zealand Clinical Trials Registry in April 2011 (ACTRN12611000402943). All participants gave their written informed consent.

The trial investigators invited 5250 people, mainly from family practices in Auckland (*n* = 5107) and a small number from community groups (*n* = 143), who underwent baseline assessments at the School of Population Health, Tamaki Campus, University of Auckland, between 5 April, 2011 and 6 November, 2012. The participants were asked questions about their demographic status, lifestyle (including smoking and physical activity), intake of vitamin D supplements within the study inclusion criteria (≤600 IU per day if aged 50–70 years; ≤800 IU per day if aged 71–84 years), and past medical history, as informed by a doctor (including asthma, COPD, and other medical disorders) [13]. The weight (to the nearest 0.1 kg) and height (to the nearest 0.1 cm) of the participants were measured in the research clinic.

Spirometry was performed using a KoKo Trek spirometer (nSpire Health; Longmont, CO, USA) with participants in a seated position, maximally inhaling and then forcibly exhaling while watching a clock on a computer screen for at least 6 s. Only three efforts were performed due to time constraints as a result of the large sample size, and to avoid exhaustion in the elderly participants. All other spirometry recommendations were fulfilled [17]. The maximum values of forced expiratory volume in 1 s (FEV1; in L), forced vital capacity (FVC; in L), and their ratio (FEV1/FVC) were derived from the three efforts.

A blood sample was collected and immediately centrifuged for an initial measurement of corrected serum calcium (those >2.50 mmol/L were excluded). The remaining serum was stored at −80 °C for later measurement of serum 25OHD, using liquid chromatography–tandem mass spectrometry (API 4000 by SCIEX; Framingham, MA, USA) with 12.7% interassay coefficient of variation, by a local laboratory participating in the Vitamin D External Quality Assessment Scheme program (www.deqas.org). The baseline 25OHD was deseasonalized using standard methods [18].

After the baseline assessment, participants were mailed a run-in questionnaire with a masked placebo capsule. On return of the questionnaire within four weeks, 5110 participants were randomized by the study statistician—within random block sizes of 8, 10, or 12, and stratified by ethnic origin (Māori, Pacific Island, South Asian, European, or other) and 5-year age groups—to receive identical-looking softgel capsules containing either vitamin D_3_ (100,000 IU) or placebo. The capsules were provided by Tishcon Corporation (Westbury, NY, USA).

The capsules were mailed monthly to the homes of the participants, with two capsules in the first letter (an initial bolus of 200,000 IU vitamin D_3_ or placebo), and thereafter, one capsule monthly (100,000 IU vitamin D_3_ or placebo). Two participants (both from the placebo arm) withdrew during the follow-up period, which ended on 31 July 2015, so that 2558 participants received vitamin D_3_ and 2550 received placebo capsules.

New Zealand residents have a unique National Health Index (NHI) number. This number was used to link each participant to information held by the Ministry of Health on all dispensed prescriptions from two years before the assessment until the end of the follow-up on 31 July 2015. This included information on the medication, dose, number of tablets or volume dispensed, and date of dispensing. Data on all medications used for the management of asthma or COPD were extracted; these included short-acting β-agonists (SABA)—inhaled or not inhaled, inhaled long-acting β-agonists (LABA), inhaled short or long-acting muscarinic antagonists (SAMA or LAMA), inhaled combination SABA and SAMA agents, inhaled mast cell stabilizers, inhaled corticosteroids (ICS) with or without LABA, and oral corticosteroids. No participants with asthma or COPD received a prescription for inhaled orciprenaline, injected bronchodilators (such as aminophylline), or injected corticosteroids. The Ministry of Health also provided information about any deaths in the cohort during the follow-up period.

Asthma identification: Participants were asked at their baseline assessment, “Have you ever been told by a doctor that you have asthma?” Those who answered “Yes” and had been dispensed a prescription for ICS, SABA, or LABA at any time from 12 months before randomization to 36 months after, were defined as having asthma and were included in the current analysis.

COPD identification: Given the well-known underdiagnosis of COPD in the general population [19,20], spirometry was used to identify participants who had a ratio of forced expiratory volume in one second (FEV_1_), divided by forced vital capacity (FVC) of <0.70, and who had smoked >100 cigarettes in their whole life, regardless of whether they were current or former smokers. These individuals were defined as having COPD, and were included in the current analysis.

Primary outcome: Exacerbations of asthma or COPD were identified by any prescription of oral corticosteroids more than 20 days apart for a short period (e.g., several days), consistent with dosing regimens recommended in international management guidelines [3,4], after joint adjudication by two senior clinicians (CAC, LT) who were blinded to the treatment allocation. Any participants selected according to the above asthma or COPD identification criteria were excluded if they had medical conditions commonly treated by oral corticosteroids—e.g., doctor-diagnosed arthritis reported at baseline, except for osteoarthritis; and doctor-diagnosed inflammatory bowel disease or multiple sclerosis reported at baseline or in the final end-of-study questionnaire in July 2015.

**Figure 1:**
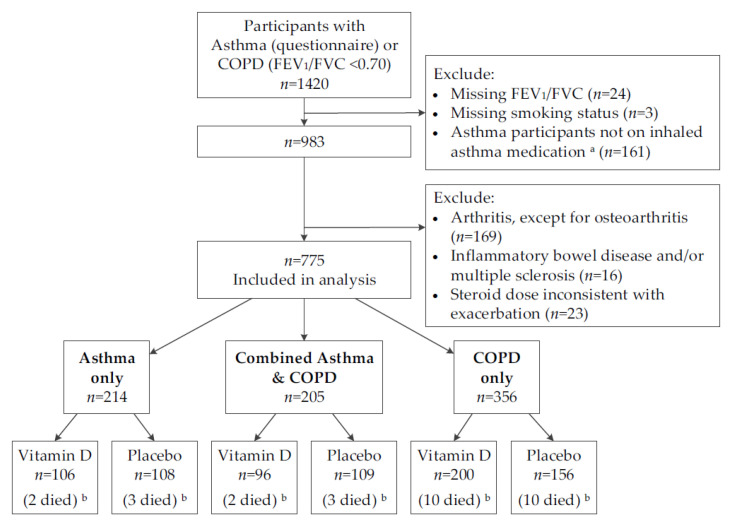
Flow diagram for selecting participants with asthma or chronic obstructive pulmonary disease in the ViDA study. COPD = chronic obstructive pulmonary disease; FEV_1_ = forced expiratory volume in 1 s; FVC = forced vital capacity; SABA = short-acting β-agonist; LABA = long-acting β-agonist. ^a^ Dispensed inhaled corticosteroids, SABA or LABA at any time from 12 months before randomisation, to 36 months after. ^b^ Died by 31 July 2015.

Figure 1 shows that 1420 participants were selected by the initial screen of either having doctor-diagnosed asthma or a FEV_1_/FVC ratio <0.70. After excluding participants with missing FEV_1_/FVC ratios, asthma participants not dispensed an inhaled asthma medication, and COPD participants that had missing information on their smoking status or those who had never been smokers, 983 participants remained eligible. Of these, a further 208 were excluded for having conditions commonly treated by oral corticosteroids. This left an analytic sample of 775 participants for the post hoc analysis, with 214 having asthma only, 356 having COPD only, and 205 having both conditions; 30 of these participants died during follow-up. This selection was made without knowledge of the treatment allocation.

### Statistical Analysis

Data were analyzed using SAS (Cary, NC, USA, version 9.4). Chi-square tests and t-tests were used to compare proportions and means, respectively. The PHREG procedure was used to calculate hazard ratios (HR) for repeated prescriptions of oral corticosteroids, using the mean cumulative function overlay for comparison groups, adjusted for covariates. The mean cumulative function is the average cumulative number of prescriptions at a time point during follow-up. Effect modification was assessed by the creation of interaction terms in models that also included the main effects for both variables in the regression model. Incidence rates were calculated by dividing the number of oral steroid prescriptions by person-time. *p*-values were not corrected to account for the multiple hypothesis tests because, given the known heterogeneity in the effectiveness of vitamin D supplementation in preventing ARI and asthma or COPD exacerbations [5,7,8], we did not want to miss any potentially important findings [21]. A two-sided *p* < 0.05 was considered statistically significant.

## 3. Results

The selected sample of participants had a mean (SD) age of 66.6 (8.3) years and 56.4% were men. Most participants were of European/Other ethnicity (82.7%) and well-educated, with 49.7% having attended tertiary education and 48.7% still in paid employment. Only 12.4% were current tobacco smokers, and a high proportion were former smokers (62.7%). Overall, the mean (SD) FEV_1_ was 2.16 (0.70) L, FVC was 3.24 (0.96) L, and ratio of FEV_1_/FVC was 0.67 (0.10); the observed baseline 25OHD was 62.5 (23.7) nmol/L.

With regard to disease severity, 74% of participants with asthma only were taking a long-term controller medication (e.g., inhaled corticosteroids) at baseline. Among all participants with COPD, the baseline mean (SD) FEV_1_ was 2.09 (0.69) L, FVC was 3.31 (0.96) L, and ratio of FEV_1_/FVC was 0.63 (0.07). Most COPD patients were in GOLD stages 1 and 2: 226 (40%) in stage 1, 271 (48%) in stage 2, 58 (10%) in stage 3, and 6 (1%) in stage 4.

Table 1 compares baseline characteristics between the vitamin D and placebo groups. There were no between-group differences in distributions of demographic or lifestyle variables, asthma or COPD status, or in mean levels of spirometry and anthropometry (*p* > 0.05). However, participants in the vitamin D group had slightly higher observed and deseasonalized mean 25OHD concentrations than those in the placebo group (*p* < 0.05).

**Figure 2:**
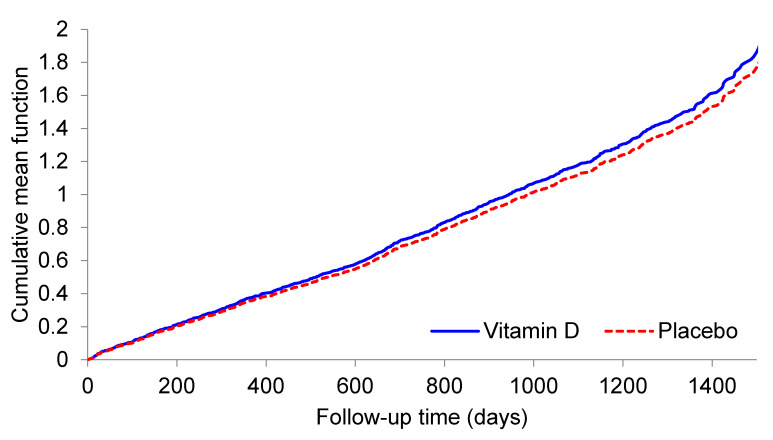
Mean cumulative number of exacerbations of asthma or chronic obstructive pulmonary disease during follow-up to 31 July 2015, by study treatment.

Table 2 shows the number and incidence rates of oral corticosteroid prescriptions for all participants, and for demographic and 25OHD categories within the vitamin D and placebo groups; it also shows HRs for repeated prescriptions in the vitamin D group compared to the placebo group, adjusted for age, sex, and ethnicity. For all participants in the analysis, the incidence rate for oral corticosteroid prescriptions was 0.40 per person-year in the vitamin D group and 0.39 per person-year in the placebo group. The HR was 1.08 (95%CI 0.84–1.39) adjusting for age, sex, and ethnicity, with the mean cumulative function curves being similar in the two treatment groups (Figure 2).

The incidence rate was lower in both treatment arms for participants with COPD only, compared to those with asthma only or with combined asthma/COPD. However, vitamin D had no effect in preventing prescriptions within each of these asthma-COPD categories, as HRs were not different from 1.00 (*p* > 0.05). Unexpectedly, the incidence rate was increased in women given vitamin D compared to placebo (HR 1.46; 95%CI 1.03–2.06; *p* for interaction = 0.04). This finding prompted a post hoc analysis to examine for possible baseline imbalance in lung function, a major predictor of exacerbations. Indeed, among women, the vitamin D group had worse lung function (e.g., FEV_1_ % predicted 71% in vitamin D group vs. 75% in placebo group; *p* = 0.09). Adjusting for this modest difference attenuated the original HR from 1.46 to 1.28 (95%CI 0.92–1.79).

**Figure 3:**
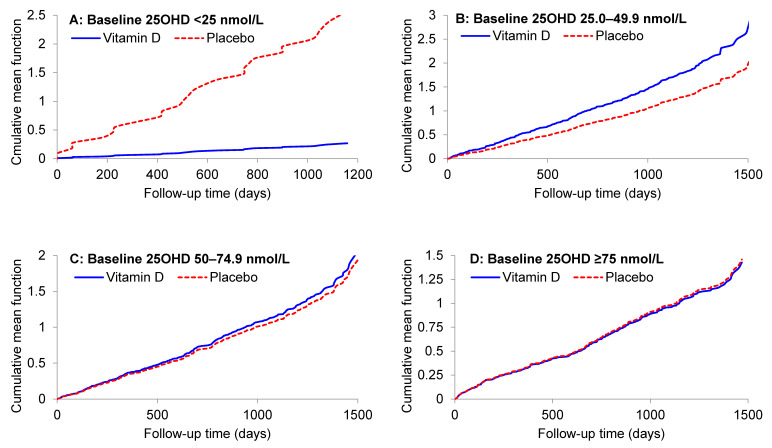
Mean cumulative number of exacerbations of asthma or chronic obstructive pulmonary disease during follow-up to 31 July 2015 for deseasonalized baseline serum 25-hydroxyvitamin D subgroups (panels **A**–**D**), by study treatment.

When participants were analyzed by baseline 25OHD category, there was a strong protective effect of vitamin D supplementation in those with deseasonalized 25OHD <25 nmol/L (HR 0.11, 95%CI 0.02–0.51; *p* = 0.005; adjusting for age, sex, and ethnicity), along with a highly significant interaction when comparing the effect of vitamin D in participants with a baseline 25OHD below and above 25 nmol/L (*p* for interaction = 0.001). Further adjustment for baseline FEV_1_ % predicted had no material impact on the effect of vitamin D in participants with baseline 25OH <25 nmol/L (HR 0.10; *p* = 0.004). The HR reduction was slightly attenuated after adjusting for asthma-COPD status (HR 0.24; *p* = 0.06), but the interaction comparing participants with baseline 25OHD below and above 25 nmol/L remained highly significant (*p* for interaction = 0.002). To explore if there were potentially important differences in the distribution of baseline variables from participants with baseline 25OHD <25 nmol/L, we repeated Table 1 in this subgroup; randomization yielded two similar groups (Supplementary Materials, Table S1). Figure 3 shows the mean cumulative function for each treatment group by baseline 25OHD category.

Adherence-related data support fidelity to the protocol. For example, 98% of the 775 participants reported taking the study capsule over the study period. In prior publications, we have documented, in the random subset of participants who underwent multiple blood testing as part of a safety evaluation, that the observed blood 25OHD levels in the intervention group increased from approximately 63 nmol/L to 135 nmol/L, consistent with their vitamin D supplementation, while the 25OHD in the placebo group did not change [14,15,16]. Participant retention was also high; for example, 77% of the 775 participants returned the final monthly questionnaire (July 2015). Lastly, we have previously reported that the vitamin D intervention did not affect participant-reported adverse events [22,23].

To provide better context for the mostly null RCT findings, we also examined the *observational* (noninterventional) association between baseline 25OHD levels and future risk of asthma or COPD exacerbation in the placebo group only (Supplementary Materials, Table S2). Participants with asthma (with or without COPD) had significantly increased HR compared to those with COPD only. The HR also was increased in older participants, men, and Māori or Pacific Island participants. Despite the limited statistical power, there was a borderline significant (*p* = 0.08) increase in HR among participants with baseline 25OHD <25 nmol/L compared to those ≥75 nmol/L.

## 4. Discussion

In this post hoc analysis of RCT data from 775 older adults with asthma or COPD, monthly high-dose vitamin D supplementation (compared to placebo) did not prevent exacerbations of asthma or COPD. However, in the prespecified subgroup with baseline 25OHD <25 nmol/L, the observed benefit was striking (HR 0.11; 95%CI 0.02–0.51). The consistency of this subgroup finding with prior RCTs focused on either asthma or COPD [7,8] suggests that the finding is not due to chance alone.

Recent IPD meta-analyses of RCTs on the effects of vitamin D supplementation on ARI [5], asthma exacerbations [7], and COPD exacerbations [8] provide an excellent overview of the most relevant RCT literature. Briefly, the ARI meta-analysis showed an overall benefit of vitamin D supplements, but with substantial heterogeneity according to study population and dosing regimen; those who received the most benefit had baseline 25OHD <25 nmol/L and did not receive bolus dosing [5]. The asthma exacerbation meta-analysis showed an overall benefit, and suggested potentially greater benefit among those with baseline 25OHD <25 nmol/L [7]. Lastly, the COPD exacerbation meta-analysis showed no benefit overall, but consistent benefit for those with baseline 25OHD <25 nmol/L [8]. The current RCT findings are consistent with the results of the asthma and COPD meta-analyses. The new findings extend the results of earlier studies by having a much longer duration (average of 3.3 years) and by showing, in the same RCT, that despite no apparent effect on ARI [16], monthly vitamin D supplementation prevents asthma/COPD exacerbations among those with vitamin D deficiency.

The apparent discordance between the effects of vitamin D supplementation on the prevention of ARI *per se* (avoid bolus dosing for benefit) versus on asthma/COPD exacerbations (bolus dosing works in specific patient populations) merits further study. The results raise the intriguing possibility that, while viral respiratory infections trigger most exacerbations, the beneficial effects of vitamin D on asthma and COPD exacerbations may involve other effects, such as its anti-inflammatory actions [24]—which may occur regardless of the exact dosing regimen. Although ViDA is underpowered to look at the effect of vitamin D supplementation among vitamin D-deficient adults with asthma only, COPD only, or asthma-COPD overlap, we look forward to contributing our data to future IPD meta-analyses. Regardless, the emerging differences in how patient population and dosing regimen can modify the effects of vitamin D supplements suggest that ARI, asthma exacerbation, and COPD exacerbation outcomes are not truly interchangeable.

Although the ViDA trial suggests that monthly high-dose vitamin D supplementation is safe, we did note a 46% elevated risk of exacerbation among women assigned to the vitamin D supplement group (Table 2). We are not aware of any prior observational or interventional study that suggests that women experience more asthma or COPD exacerbations at higher levels of circulating 25OHD, or in response to taking vitamin D supplements [6]. Accordingly, we believe these results are most likely due to chance. We encourage further research to further investigate this subgroup finding.

The current report has several major strengths, including its study design (randomized, double-blinded, placebo-controlled trial) with high protocol adherence and the clearly demonstrated effect of the intervention on blood 25OHD levels [14,15,16]. Nevertheless, this RCT, like all RCTs, has potential limitations. First, we remind readers that we tested one vitamin D regimen (initial bolus, then monthly high-dose boluses) in one population (older adults); therefore, the relevance of the current study to, for example, daily vitamin D dosing in children with asthma is unclear. Second, the primary outcome was based on the prescribing of systemic corticosteroids, which requires clinician recognition of the exacerbation and prescribing of the appropriate treatment. While it is likely that we missed exacerbation events, we assume that these events were equally distributed across the two randomly assigned groups; this would tend to bias results toward the null. Third, despite starting with 775 participants, the few trial participants with baseline 25OHD <25 nmol/L (*n* = 18, 2%) precluded analyses within the asthma only, COPD only, or asthma-COPD overlap groups. By contributing our trial data to future IPD meta-analyses, we hope to compile sufficiently large numbers of participants in these important patient groups to pursue the issues further.

In summary, although monthly high-dose vitamin D supplementation had no overall impact on exacerbations of asthma or COPD in these older adults, we found evidence of probable benefit among those with severe vitamin D deficiency (baseline 25OHD < 25 nmol/L). While it remains possible that the subgroup finding was due to chance, it was very similar to results from other vitamin D trials in the literature, particularly those focused on the prevention of COPD exacerbations [8]. The exact threshold (and mechanism) for the observed benefit requires further study. To maximize the scientific yield of future RCTs, we encourage that vitamin D researchers carefully consider the importance of the trial population, vitamin D dosing regimen, and other factors when both designing and interpreting the current results, and those from future trials [25].

## Figures and Tables

**Table 1:** Baseline comparison of the vitamin D supplemented and placebo groups.

Variable	Vitamin D (*n* = 402)	Placebo (*n* = 373)	*p*-Value
Age (years)			0.77
50–59	84 (20.9)	75 (20.1)	
60–69	169 (42.0)	152 (40.8)	
70–79	128 (31.8)	120 (32.2)	
80–84	21 (5.2)	26 (7.0)	
Sex–male	227 (56.5)	210 (56.3)	0.96
Ethnicity			0.25
Māori	30 (7.5)	38 (10.2)	
Pacific Island	15 (3.7)	22 (5.9)	
South Asian	16 (4.0)	13 (3.5)	
European/Other	341 (84.8)	300 (80.4)	
Education (highest level) ^a^			0.71
Primary school	8 (2.0)	5 (1.3)	
Secondary school	198 (49.3)	179 (48.0)	
Tertiary	196 (48.8)	189 (50.7)	
In paid employment ^a^			0.45
Yes	203 (50.5)	174 (46.8)	
No			
Retired	172 (42.8)	166 (44.6)	
Other	27 (6.7)	32 (8.6)	
Tobacco smoking			0.95
Current	50 (12.4)	46 (12.3)	
Ex	250 (62.2)	236 (63.3)	
Never	102 (25.4)	91 (24.4)	
Vigorous physical activity (hours per week) ^a^			0.09
None	149 (38.6)	163 (46.4)	
1–2	102 (26.4)	76 (21.7)	
>2	135 (35.0)	112 (31.9)	
Take vitamin D supplements ^b^	25 (6.2)	31 (8.3)	0.26
Type of asthma/COPD			0.07
Asthma only	106 (26.4)	108 (29.0)	
COPD only	200 (49.8)	156 (41.8)	
Combined asthma & COPD	96 (23.9)	109 (29.2)	
Spirometry, mean (SD)			
FEV_1_ (L)	2.18 (0.71)	2.15 (0.70)	0.53
FEV_1_, % predicted	78 (19)	79 (19)	0.98
FVC (L)	3.27 (0.98)	3.21 (0.93)	0.37
FVC, % predicted	90 (17)	91 (17)	0.79
Ratio FEV_1_/FVC	0.67 (0.09)	0.67 (0.10)	0.69
FEV_1_/FVC, % predicted	86 (12)	86 (11)	0.70
PEF (L/min)	353 (116)	360 (118)	0.43
Anthropometry, mean (SD)			
Height (cm)	168.9 (9.3)	168.6 (9.2)	0.70
Weight (kg)	80.3 (16.2)	80.6 (17.3)	0.81
Body mass index (kg/m^2^)	28.1 (5.1)	28.3 (5.6)	0.57
Corrected serum calcium, mean (SD) (mmol/L)	2.28 (0.07)	2.27 (0.07)	0.10
25-hydroxyvitamin D (nmol/L)			
Observed, mean (SD)	64.5 (23.1)	60.4 (24.2)	0.02
Deseasonalized, mean (SD)	66.7 (22.3)	63.1 (22.9)	0.03
Deseasonalized category			0.03
<25.0	8 (2.0)	10 (2.7)	
25.0–49.9	82 (20.4)	109 (29.2)	
50.0–74.9	172 (42.8)	134 (35.9)	
≥75.0	140 (34.8)	120 (32.2)	

Results are number (%) unless otherwise indicated. Abbreviations: FEV_1_ = forced expiratory volume in 1 s; FVC = forced vital capacity; PEF = peak expiratory flow rate. ^a^ Numbers do not add to total for column because of missing/don’t know responses. ^b^ ≤600 IU per day if aged 50–70 years; ≤800 IU per day if aged 71–84 years.

**Table 2:** Numbers, incidence rates, and adjusted hazard ratios of oral corticosteroid prescriptions (placebo as reference), by treatment group.

Participants	Vitamin D			Placebo			Hazard Ratio (95%CI) ^a^	*p*-Value (Wald X^2^)
	Number of Participants	Number of Prescriptions /*p*-Y	Incidence Rate per *p*-Y	Number of Participants	Number of Prescriptions /*p*-Y	Incidence Rate per *p*-Y		
All	402	536/1324	0.40	373	469/1218	0.39	1.08 (0.84, 1.39)	0.55
Asthma-COPD category								
Asthma only	106	172/352	0.49	108	173/367	0.47	1.18 (0.80, 1.74)	0.41
COPD only	200	176/650	0.27	156	124/494	0.25	1.07 (0.69, 1.67)	0.75
Combined asthma & COPD	96	188/320	0.59	109	172/357	0.48	1.21 (0.78, 1.88)	0.39
Age (years)								
50–59	84	155/299	0.52	75	117/270	0.43	1.23 (0.73, 2.07)	0.43
60–69	169	220/550	0.40	152	206/494	0.42	0.99 (0.68, 1.45)	0.97
70–79	128	139/410	0.34	120	108/374	0.29	1.21 (0.73, 2.03)	0.46
80–84	21	22/65	0.34	26	38/80	0.48	0.54 (0.19, 1.52)	0.24
Sex								
Male	227	242/744	0.33	210	270/676	0.40	0.83 (0.58, 1.18)	0.30
Female	175	294/580	0.51	163	199/542	0.37	1.46 (1.03, 2.06)	0.03
Ethnicity								
Māori	30	41/100	0.41	38	72/127	0.57	0.68 (0.33, 1.42)	0.31
Pacific Island	15	48/53	0.91	22	49/73	0.67	1.37 (0.56, 3.31)	0.49
South Asian	16	33/51	0.65	13	20/43	0.47	1.73 (0.51, 5.92)	0.38
European/Other	341	414/1120	0.37	300	328/976	0.34	1.10 (0.82, 1.46)	0.54
25OHD category (nmol/L) ^b^								
<25.0	8	2/27	0.07	10	23/33	0.70	0.11 (0.02, 0.51)	0.005
25.0–49.9	82	152/265	0.57	109	150/360	0.42	1.45 (0.92, 2.30)	0.11
50.0–74.9	172	232/569	0.41	134	168/437	0.38	1.09 (0.76, 1.58)	0.64
≥75.0	140	150/463	0.32	120	128/389	0.33	1.03 (0.62, 1.70)	0.92

Abbreviations: 25OHD = 25-hydroxyvitamin D; 95%CI, 95% confidence interval; COPD = chronic obstructive pulmonary disease; *p*-Y = person-years. ^a^ Adjusted for age, sex, and ethnicity (as appropriate). ^b^ Based on deseasonalized concentrations.

## Data Availability

The data presented in this study are available on reasonable request from the corresponding author. The data are not publicly available due to privacy restriction.

## Supplementary Materials

The following tables are available online at https://www.mdpi.com/2072-6643/13/2/521/s1: Table S1: Baseline comparison of the vitamin D supplemented and placebo groups among participants with 25-hydroxyvitamin D < 25 nmol/L. Table S2: Hazard ratios of oral corticosteroid prescriptions, adjusted for other variables in the table, in the placebo group only (*n* = 373).
