# Supplementary material for: Effect of Monthly Vitamin D Supplementation on Preventing Exacerbations of Asthma or Chronic Obstructive Pulmonary Disease in Older Adults: Post Hoc Analysis of a Randomized Controlled Trial
Source: Nutrients. 2021 Feb 6;13(2):521. doi: 10.3390/nu13020521 (PMC7915442; doi:10.3390/nu13020521)
Supplement: Supplementary file 1 [file nutrients-13-00521-s001.pdf]

ONLINE SUPPLEMENT

Effect of monthly vitamin D supplementation on preventing exacerbations of asthma or chronic obstructive pulmonary disease in older adults: *post hoc* analysis of a randomized controlled trial

Carlos A. Camargo Jr., MD DrPH [1,*], Les Toop, MBChB MD [2], John Sluyter, PhD [3], Carlene M.M. Lawes, MBChB PhD [3], Debbie Waayer, MEd [3], Kay-Tee Khaw, MBBChir MSc [4], Adrian R Martineau, MBBS PhD [5] and Robert Scragg, MBBS PhD [3]

[1] Department of Emergency Medicine, Massachusetts General Hospital, Harvard Medical School, Boston, USA; ccamargo@partners.org

[2] Department of General Practice, University of Otago, Christchurch, New Zealand; les.toop@otago.ac.nz

[3] School of Population Health, University of Auckland, Auckland, New Zealand; j.sluyter@auckland.ac.nz, Carlene.Lawes@waitematadhb.govt.nz, d.waayer@auckland.ac.nz, and r.scragg@auckland.ac.nz

[4] Department of Public Health and Primary Care, University of Cambridge, Cambridge, UK; kk101@cam.ac.uk

[5] Institute for Population Health Sciences, Barts and The London School of Medicine and Dentistry, Queen Mary University of London, London, UK; a.martineau@qmul.ac.uk

* Correspondence: ccamargo@partners.org

TABLE OF CONTENTS

- **Table S1.** Baseline comparison of the vitamin D supplemented and placebo groups among participants with 25-hydroxyvitamin D <25 nmol/L.

- **Table S2:** Hazard ratios for exacerbations of asthma or chronic obstructive pulmonary disease, adjusted for other variables in the table, in the placebo group only (n=373).

Table S1. Baseline comparison of the vitamin D supplemented and placebo groups among participants with 25-hydroxyvitamin D <25 nmol/L.

Variable	Vitamin D (n=8)	Placebo (n=10)	P-value
Age (years)			
50-59	3 (37.5)	2 (20.0)	
60-69	2 (25.0)	4 (40.0)	
70-79	2 (25.0)	4 (40.0)	0.64
80-84	1 (12.5)	0 (0.0)	
Sex – male	6 (75.0)	6 (60.0)	0.32
Ethnicity			
Māori	0 (0.0)	0 (0.0)	
Pacific Island	0 (0.0)	2 (20.0)	
South Asian	3 (37.5)	3 (30.0)	0.64
European / Other	5 (62.5)	5 (50.0)	
Education (highest level) [a]			
Primary school	0 (0.0)	1 (10.0)	
Secondary school	3 (37.53)	4 (40.0)	1.00
Tertiary	5 (62.5)	5 (50.0)	
In paid employment [a]			
Yes	2 (20.0)	4 (50.0)	
No			
Retired	6 (60.0)	3 (37.5)	0.46
Other	2 (20.0)	1 (12.5)	
Tobacco smoking			
Current	1 (12.5)	2 (20.0)	
Ex	5 (62.5)	4 (40.0)	0.82
Never	2 (25.0)	4 (40.0)	
Vigorous physical activity (hours per week) [a]			
None	3 (37.5)	7 (77.8)	
1-2	2 (25.0)	1 (11.1)	
>2	3 (37.5)	1 (11.1)	0.33
Take vitamin D supplements [b]	0 (0.0)	0 (0.0)	-
Type of asthma/COPD			
Asthma only	2 (25.0)	5 (30.0)	
COPD only	4 (50.0)	2 (20.0)	0.43
Combined asthma & COPD	2 (25.0)	3 (30.0)	
Spirometry, mean (SD)			
FEV_1 (L)	2.07 (0.43)	1.87 (0.70)	0.48
FEV_1, % predicted	74 (14)	73 (24)	0.94

FVC (L)	3.02 (0.71)	2.80 (0.94)	0.59
FVC, % predicted	84 (14)	85 (24)	0.87
Ratio FEV_1/FVC	0.69 (0.07)	0.68 (0.16)	0.86
FEV_1/FVC, % predicted	88 (7)	86 (18)	0.75
PEF (L/min)	357 (108)	301 (132)	0.35
Anthropometry, mean (SD)			
Height (cm)	170.0 (10.4)	165.3 (9.1)	0.33
Weight (kg)	83.2 (25.8)	73.2 (19.8)	0.37
Body mass index (kg/m^2)	29.3 (11.0)	26.5 (5.2)	0.48
Corrected serum calcium, mean (SD) (mmol/L)	2.36 (0.09)	2.31 (0.14)	0.35
25-hydroxyvitamin D (nmol/L)			
Observed, mean (SD)	22.3 (9.7)	16.6 (8.8)	0.24
Deseasonalized, mean (SD)	21.8 (3.1)	18.5 (4.5)	0.12

Results are number (%) unless otherwise indicated.

Abbreviations: BMI = body mass index; FEV_1 = forced expiratory volume in 1 second; FVC = forced vital capacity; PEF = peak expiratory flow rate.

[a] Numbers do not add to total for column because of missing/don't know responses.

[b] ≤600 IU per day if aged 50-70 years; ≤800 IU per day if aged 71-84 years.

Table S2: Hazard ratios for exacerbations of asthma or chronic obstructive pulmonary disease, adjusted for other variables in the table, in the placebo group only (n=373).

**Participants**	**Hazard Ratio (95%CI) [a]**	**P-value (Wald X^2)**	**P-value (Wald X^2)**
Asthma-COPD category			<0.0001
Asthma only	1.96 (1.45, 2.66)	<0.0001	
COPD only	1.00	-	
Combined Asthma & COPD	2.10 (1.59, 2.77)	<0.0001	
Age (years)			0.005
50-59	1.00	-	
60-69	1.37 (1.01, 1.84)	0.04	
70-79	0.95 (0.67, 1.34)	0.76	
80-84	1.85 (1.14, 3.01)	0.01	
Sex			0.003
Male	1.44 (1.14, 1.83)	0.003	
Female	1.00	-	
Ethnicity			0.0003
Māori	1.82 (1.26, 2.64)	0.002	
Pacific Island	2.15 (1.44, 3.22)	0.0002	
South Asian	1.08 (0.59, 1.98)	0.80	
European / Other	1.00	-	
25OHD category (nmol/L) [b]			0.38
< 25.0	1.70 (0.94, 3.07)	0.08	
25.0 – 49.9	1.06 (0.78, 1.43)	0.72	
50.0 – 74.9	1.04 (0.78, 1.39)	0.79	
≥ 75.0	1.00	-	

Abbreviations: 25OHD, 25-hydroxyvitamin D; 95%CI, 95% confidence interval; COPD, chronic obstructive pulmonary disease.

[a] Adjusted for all other variables in the table

[b] Based on deseasonalized concentrations
